# Variations in the Density and Distribution of Cajal Like Cells Associated With the Pathogenesis of Ureteropelvic Junction Obstruction: A Systematic Review and Meta-Analysis

**DOI:** 10.3389/fsurg.2021.721143

**Published:** 2021-07-28

**Authors:** U. M. J. E. Samaranayake, Y. Mathangasinghe, U. A. Liyanage, M. V. C. de Silva, M. C. Samarasinghe, S. Abeygunasekera, A. K. Lamahewage, A. P. Malalasekera

**Affiliations:** ^1^Department of Anatomy, Faculty of Medicine, Sabaragamuwa University of Sri Lanka, Ratnapura, Sri Lanka; ^2^Department of Anatomy, Faculty of Medicine, University of Colombo, Colombo, Sri Lanka; ^3^Proteostasis and Neurodegeneration Laboratory, Australian Regenerative Medicine Institute, Monash University, Clayton, VIC, Australia; ^4^Department of Pathology, Faculty of Medicine, University of Colombo, Colombo, Sri Lanka; ^5^Department of Surgery, Faculty of Medicine, University of Colombo, Colombo, Sri Lanka; ^6^Lady Ridgeway Hospital for Children, Colombo, Sri Lanka

**Keywords:** interstitial cells of Cajal, Cajal like cells, ureteropelvic junction obstruction, density, aging

## Abstract

**Introduction:** Cajal like cells (CLCs) in the upper urinary tract have an ability to generate coordinated spontaneous action potentials and are hypothesized to help propel urine from renal pelvis into the ureter. The objective of this review was to describe the variations in the density and distribution of CLCs associated with ureteropelvic junction obstruction (UPJO).

**Materials and Methods:** Studies comparing the density and distribution of CLCs in the human upper urinary tract in patients with UPJO and healthy controls were included in this systematic review. We searched online electronic databases; Ovid MEDLINE, Scopus, PubMed and Cochrane reviews for the studies published before October 31, 2020. A meta-analysis was conducted to compare the density of CLCs at the ureteropelvic junction (UPJ) in patients with UPJO and matched controls.

**Results:** We included 20 and seven studies in the qualitative and quantitative synthesis, respectively. In majority (55%) CLCs were located between the muscle layers of the upper urinary tract. The CLC density in the UPJ gradually increased with aging in both healthy subjects and patients with UPJO. The pooled analysis revealed that the density of CLCs at the UPJ was significantly low in patients with UPJO compared to the controls (SMD = −3.00, 95% CI = −3.89 to −2.11, *p* < 0.01).

**Conclusions:** The reduction in CLC density at the UPJ in patients with UPJO suggests a contribution from CLCs in the pathogenesis of UPJO. Since age positively correlates with CLC density, it is imperative to carefully match age when conducting case control studies comparing the CLC density and distribution.

**Protocol Registration Number:** CRD42020219882.

## Introduction

Primary ureteropelvic junction obstruction (UPJO) is the most common congenital abnormality causing hydronephrosis in children (1) which affects 1 in 750–1,500 newborns annually (2–4). Structurally, the UPJO is characterized by a narrowed segment of the ureteropelvic junction (UPJ) containing atrophied smooth muscles and a hypertrophied segment proximal to the obstruction with increased collagen deposition (5). The widely accepted theory for the pathogenesis of UPJO is the disruption of coordinated unidirectional smooth muscle contractions, leading to dampening of peristaltic waves that propels urine downward from the renal pelvis to the ureter (6). Nevertheless, the exact mechanism of how these unidirectional contractions are coordinated in healthy ureteropelvic junction remains a mystery. Nearly a century ago Santiago Ramón y Cajal discovered a cell, later named in his honor, which has a regulatory role in smooth muscle contractility. These cells form a plexus that runs between the gut muscle layers, with processes extending from the ganglion cells of Auerbach plexus and nerve terminals residing on the plasmalemma of smooth muscle cells (7). These cells express c-kit (CD177) encoding receptor tyrosine kinase in their cytoplasmic membrane, which allow visualization of them using immunostaining (8). Reduction in the density of intestinal Cajal cells was later found to be associated with motility disorders of the gastrointestinal system such as congenital pyloric stenosis, achalasia cardia, Hirschsprung's disease and chronic intestinal pseudo obstruction (9–12).

Huizinga and Faussone-Pellegrini (13) reported the presence of different subtypes of Cajal cells, termed Cajal like cells (CLCs), outside the gastrointestinal tract with unique ultrastructural characteristics that help distinguish them from other cell types expressing c-kit such as mast cells, glial cells and melanocytes. The CLCs in the urinary tract have a stellate shape or a fusiform cell body with two distinct dendrites (14, 15). Subsequently, CLCs were identified in many organs including urinary tract, vagina, blood vessels and glands (13, 16, 17). The CLCs in the upper urinary tract in guinea pigs generate and amplify action potentials both in the renal pelvis and the ureter (18, 19), suggesting a unique role of CLCs in maintaining a unidirectional flow of urine at the UPJ (20, 21). With the discovery of an intrinsic motility action of the human UPJ (20), the CLCs were considered to be the pacemaker regulating the expulsion of urine at the UPJ. Nonetheless, the postulated role of CLCs in the pathogenesis of UPJO was challenged since the early studies failed to demonstrate a consistent decrease in the density of the CLCs at the UPJ in patients with UPJO (22, 23). These contradicting results led to further studies that primarily focused on the functions of the CLCs which generated clues on the pathogenesis of UPJO.

Despite decades of research, the exact pathogenic mechanism (s) of primary UPJO remains enigmatic. In this review, we provide a comprehensive analysis of the density and distribution of CLCs in the upper urinary tract associated with the UPJO and mechanistic insights to the pathophysiology of this disease. Moreover, we critically evaluate the methodological inaccuracies of certain studies which may have led to false assumptions regarding the association of the density of the CLCs at the UPJ with the UPJO.

## Materials and Methods

### Protocol and Registration

We conducted a systematic review and meta-analysis according to the Preferred Reporting Items for Systematic Reviews and Meta-Analyses (PRISMA) guidelines (24). The study protocol was documented in advance in the International Prospective Register of Systematic Reviews (PROSPERO) online database (protocol registration number: CRD42020219882).

### Eligibility Criteria

Studies comparing the density and/or distribution of CLCs in the human upper urinary tract in patients with UPJO and controls were included in this systematic review. Only the studies comparing the density of CLCs at the UPJ in patients with UPJO and matched controls were included in the quantitative synthesis. Case reports and animal studies on CLCs were excluded.

### Information Sources and Search Strategy

We searched online electronic databases; Ovid MEDLINE (Medical Literature Analysis and Retrieval System), Scopus, PubMed and Cochrane reviews. To obtain additional information, we conducted a manual search of the reference list of the selected articles. The online search strategy was generated by YM. The search comprised of studies listed up to October 31, 2020. We did not set search limits. The PubMed search strategy is provided in Table 1.

**Table 1:** The PubMed search strategy.

	**Search string**
1	Ureteropelvic
2	Pelviureteric
3	Pyeloureteric
4	Kidney
5	Urology*
6	Disease, urinary tract*
7	Ureter
8	Ureteral obstruction*
9	Interstitial cell of Cajal like cell*
10	Interstitial Cells of Cajal*
11	Telocytes*
12	1 or 2 or 3 or 4 or 5 or 6 or 7 or 8
13	9 or 10 or 11
14	12 and 13

MeSH terms are indicated by asterisks (*).

### Study Selection

Two independent reviewers (US and YM) assessed the eligibility in an unbiased standardized manner. A third reviewer (AM) was involved in case of any disagreements. We screened the total hits obtained by reading “title” and “abstract.” We excluded studies that failed to satisfy the inclusion criteria at this stage. Next, we read the full text of each selected paper to extract data. All relevant articles published in languages other than English were translated into English language before screening. The reviewers determined the final group of articles to be included in the review after an iterative consensus process.

### Data Collection Process

We developed a data extraction sheet, pilot-tested it on three randomly selected studies that were consistent with the inclusion criteria and revised it accordingly. One reviewer (US) extracted data from the included studies using this standardized form and another reviewer (YM) checked for the accuracy of data extraction. We extracted the following data from each study: (a) study details (author, country and year published), (b) sample characteristics (age of the study population and sample size), (c) methods (detection and/or quantification of CLC distribution and density) and (d) results (distribution of CLCs in a cross section and along the upper urinary tract and the density of CLCs with its association with disease status (UPJO vs. healthy subjects), age and postoperative outcomes). Ureteropelvic junction was defined as the junction between the renal pelvis and the ureter (20). Despite no clear external feature to locate the UPJ (20, 25), the internal appearance of crowding of mucosal folds forming characteristic “mucosal rosettes” allows its precise localization (20), whereas pathological UPJs in patients with UPJO is visualized intra-operatively as a valve-like appearance (26) preceding a narrowed segment with interrupted development of circular muscle fibers (27). Distribution of CLCs was defined as the location of the CLCs in different layers in the cross section of the ureter or along the upper urinary tract (UPJ, renal pelvis, or ureter). Density was defined as the total number of CLCs per high power field of an optical microscope. We resolved discrepancies in the extracted data by discussion, involving a third reviewer (AM) when necessary. We contacted the corresponding authors of the published manuscripts to obtain additional data such as the age distribution of their study populations and data sets of the measurements.

### Risk of Bias in Individual Studies

The methodological quality and the risk of bias of the included studies were assessed independently by two authors (US and YM) using Joanna Briggs Institute (JBI) Critical Appraisal Tool (28). Each criterion was evaluated as “Yes,” “No,” or “Other” (unclear/ not applicable). Overall rating was provided for each study based on the items rated with an affirmative answer and accordingly, the quality score was determined by the range 67–100 (good), 34–66 (average), and 0–33 (bad). The studies meeting the “good” scores were selected for the review.

### Quantitative Analysis

We conducted a meta-analysis of studies comparing the density of CLCs at the UPJ in patients with UPJO and matched control. A random effects model was used for the comparisons. Heterogeneity was assessed using the χ^2^ test on Cochrane's Q statistic and by *I*^2^ statistic. The *I*^2^ statistic was interpreted as follows: 0–40% might not be important; 30–60% may represent moderate heterogeneity; 50–90% may represent substantial heterogeneity; and 75–100% may represent considerable heterogeneity (29). When appropriate, sensitivity analyses were performed based on the sample size and the age distribution of the study samples to explore the sources of heterogeneity. Data were analyzed using RevMan version 5.4.1 (30). *p* < 0.05 was considered statistically significant in all analyses.

## Results

**Figure 1:**
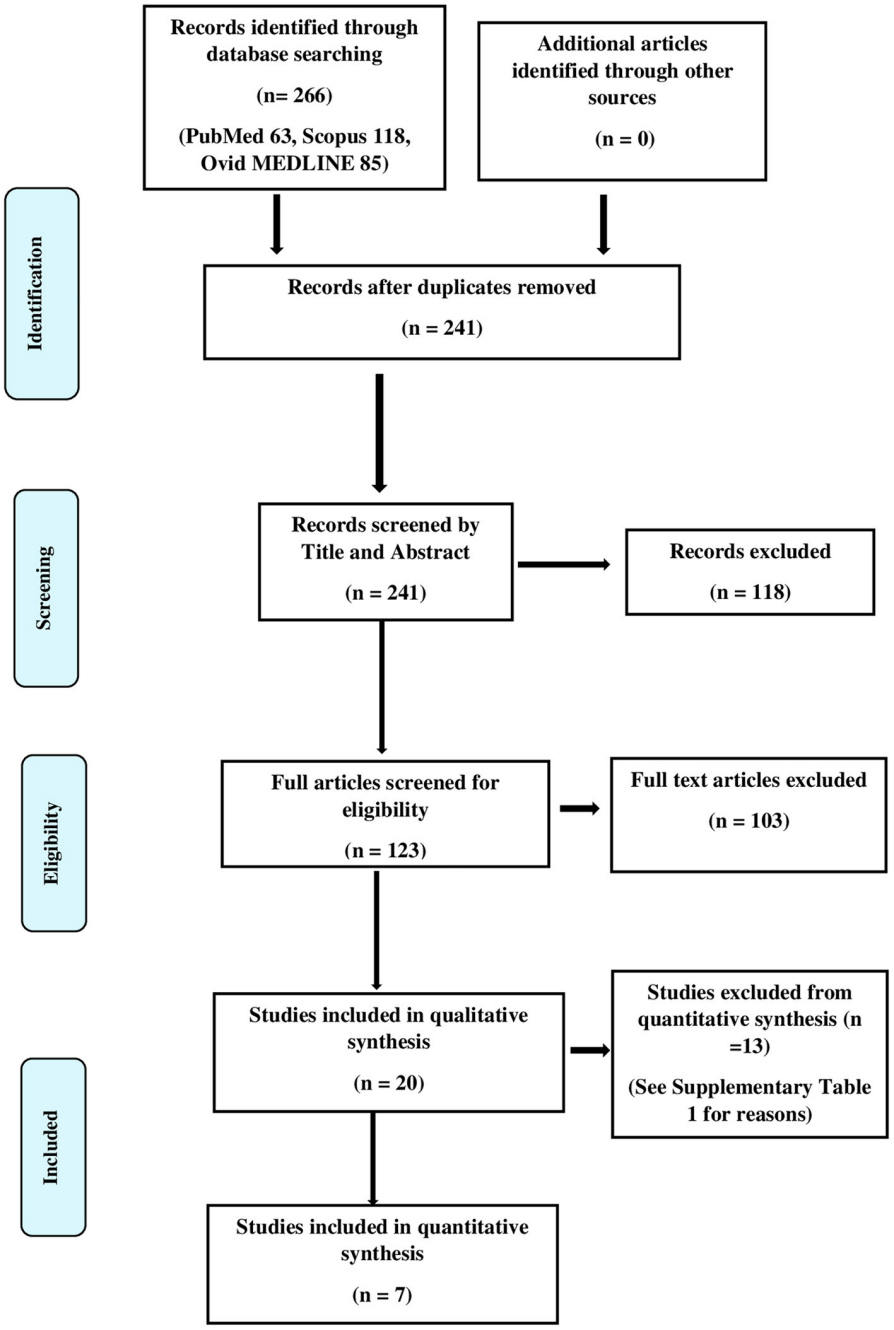
Flowchart of the study selection.

We found a total of 266 hits in the initial literature search, and after 50 duplicates were removed, 241 articles remained. We did not find additional articles after manual screening. We obtained full texts that had potential for the final review and included twenty of these studies in the final qualitative synthesis. Figure 1 illustrates the PRISMA flow diagram of the search. The results of the qualitative synthesis are summarized in Table 2. Of them, five studies presented the density of CLC as an ordinal variable (e.g., low, medium, and high density) as opposed to a continuous variable *viz*, the absolute number of CLCs per high power field, hence were subsequently excluded from the quantitative synthesis. The reasons of excluding articles from the quantitative synthesis are provided in the Supplementary Table 1. The risk of bias assessment is provided in the Supplementary Tables 2, 3. Of the studies included in the qualitative synthesis, eleven were conducted exclusively among children, while eight pooled results of adults and children. One study did not provide the age distribution of the subjects. The studies were conducted in Turkey, Poland, India, Egypt, Belgium, Germany, Korea, Singapore, Iran, Romania, Ireland, and China.

**Table 2:** Summary of the studies included in the qualitative synthesis.

	**Number of cases: control**	**Mean (± SD or range) age of cases: controls**	**Method of identification of CLCs**	**Area of CLCs distribution in ureter/UPJ**	**CLC density or distribution in cases**	**CLC density or distribution in controls**	**Conclusion of the study**
Apoznanski et al., Poland (1)	7: 5	2.2 (0.7–5.2) years: 2.3 (0.2–7.4) years	CLC gradient at the UPJ in patients with UPJO and controls was compared using IHC. Eleven adjacent HPFs (400X magnification) were examined to determine the CLC gradient. Gradient was defined as a difference of cell number per HPF greater than one in adjacent fields. Gradient was analyzed in relation to the patient's age.	Inner border of muscle layer	CLC gradient at UPJ = 19 (*P* = 0.087, *r* = −0.3927)	CLCs gradient at UPJ = 10 (*P* = 0.3753, *r* = 0.1689)	No statistically significant difference in CLC distribution between cases and controls. No correlation between age of cases and the distribution of CLCs.
Babu et al., India (15)	31: 31	2.9 (±0.6) years	The difference of CLC density of the UPJ and the anastomotic end of ureter in children with UPJO undergoing pyeloplasty was analyzed. Association between post-operative outcome of the patients and the CLC density using IHC in 10 HPF (400X magnification) under light microscope was explored.	Not available	CLC density was significantly lower in the UPJ (mean = 5.3, SD = 2.3) compared to the anastomosed end of the ureter (mean = 12.4; SD = 5.1).		UPJ had a lower density of CLCs compared to the anastomotic end of the ureter in children with UPJO undergoing pyeloplasty. Resected ureter end with mean CLC density more than 10 per HPF had a better surgical outcome.
Babu et al., India (31)	31: 20	2.9 (±3.1) years: 4.9 (±4.1) years	CLC density at the narrowed segment in patients with UPJO and controls (UPJ segments obtained from patients undergoing nephrectomy) was compared using IHC (400X magnification). The correlation between CLC density at the UPJ of the normal fetuses (aborted due to maternal conditions or intrauterine death) and gestational age was explored.	Not available	Median CLC density of the narrowed UPJO segment per HPF was 5.1 (SD = 2.3).	Median CLC density of the normal ureter per HPF was 16.1 (SD = 8.3)Median CLC density of the fetal ureter per HPF was 5.0 (SD = 2.3)	CLC density at the narrowed segment in patients with UPJO was lower than that of the normal ureter. A positive correlation was found between the increasing gestational age and the CLC density (*r* = 0.83; *P* < 0.001) in the fetal ureter.
Balikci et al., Turkey (32)	63: 30	43.5 (2–72) years: 58.6 (38–82) years	Samples were obtained from multiple areas of the urinary tract in patients with hydronephroureter due to ureteric obstruction and controls. The CLC density was studied using IHC (400X magnification) at: renal pelvis lamina propria (RPLP), renal pelvis muscularis propria (RPMP), proximal ureter lamina propria (PULP), proximal ureter muscularis propria (PUMP).	Lamina propria and muscularis propria	CLCs density in; RPLP = 22(14–28) RPMP = 26(15–36) PULP = 12(9–20) PUMP = 17(10–23)	CLCs density in; RPLP = 32(26–42) RPMP = 42(34–64) PULP = 24(20–26) PUMP = 29(25–32)	CLC density in the renal pelvis and proximal ureter in cases was significantly low (*P* < 0.001) compared to the controls.
Eken et al., Turkey (33)	35: 7	3 (0.25–18) years: 29 (10–40) years (for light microscope)	CLC density at the UPJ in patients with UPJO and controls was compared using IHC and electron microscopy. CLC density per HPF (400X magnification) was graded as: 0–3 cells/HPF = sparse 4-8 cells/HPF = few >8 cells/HPF = many	Lamina propria and muscular layer	CLC density; Sparse = 8 (22.9%) Few = 26 (74.3%) Many = 1 (2.9%)	CLC density; Sparse = 0 Few = 0 Many = 7 (100%)	CLC density was significantly higher in the controls compared to cases (*P* < 0.001). CLCs of patients with UPJO had decreased number of mitochondria and caveolae compared to controls.
How et al., Singapore (34)	38: 20	2.1 (0.2–14) years: 4.0 (0.1–16) years	Level of CD117 staining was at the UPJ in patients with UPJO and controls was compared (400X and 100X magnifications)	Not available	Difference between cases and controls with CD117 staining; No difference in cases 30 (78.9%), Increased staining in cases 8 (21.1%), Decreased staining in cases 0 (0%).		There was no statistically significant difference of CD117 staining between cases and controls.
Inugala et al., India (35)	23: 2	1.1 (0.04–4) years: 0.6 years	The association between outcome of Anderson-Hynes pyeloplasty and CLC density in resected margin was assessed. CLC density per HPF (400X magnification) was graded as: 0–1 cell/HPF = negative 2–5 cells/HPF = + 6–10 cells/HPF = ++ >11 cells/HPF = +++	Not available	CLC density at the UPJ in patients with good surgical outcomes: 0–1 cells = 12 (52.2%) 2–5 cells = 2 (8.7%) 6–10 cells = 7 (30.4%) >11 cells = 2 (8.7%) CLC density at the UPJ in patients with poor surgical outcomes: 0–1 cells = 1 (50%) 2–5 cells = 1 (50%) 6–10 cells = 0 >11 cells = 0		Having a high density of CLCs at the resection margin was associated with good surgical outcomes (*p* = 0.001).
Kart et al., Turkey (36)	11: 7	3.9 (±2.6) years: 3.6 (±3.8) years	CLC density at the UPJ in patients with UPJO and controls was compared using IHC (400X magnification)	Between Muscle layers	CLC density in cases per HPF was 1.75 (SD = 1.14)	CLC density in controls per HPF was 5.76 (SD = 2.99)	CLC density was significantly lower in cases compared to controls (*P* < 0.01).
Koleda et al., Poland (23)	20: 5	8.1 (0.7–16.8) years: 2.3 (0.2–7.4) years	CLC density at the UPJ in patients with UPJO and controls was compared using IHC. CLC density per HPF (400X magnification) was graded as: few (0 to 1), moderate (2 to 3), many (4 to 8) cells. The correlation between CLC density and age of the patients was explored.	Not available	Number of fields with few CLCs was significantly lower in cases than in controls (*P* = 0.0122). The number of fields with many CLCs was significantly higher in cases than in controls (*P* = 0.0004).		CLC density was significantly higher in cases compared to controls. CLC density of patients with UPJO decreased with aging (*r* = −0.6167, *P* = 0.0038).
Kuvel et al., Turkey (22)	32: 30	Not available	CLC density at the UPJ in patients with UPJO and controls was compared using IHC (400X magnification) Cases were classified according to location of sample obtained from the UPJ; Group Ia (proximal) Group Ib (intermediate) Group Ic (distal) segments	lamina propria (LP), muscularis propria (MP), and serosa (S) layers	CLC density in Group Ia was higher than Group Ib for LP, MP and for S layers. Group Ic had increased CLCs in LP and MP.	CLCs density in Group Ia was increased compared to controls for LP (*p* < 0.05) and S (*p* < 0.01).In intrinsic UPJO, CLCs were located more in LP and S compared to chronic UPJO.	An increased density of CLCs was observed in proximal segments of UPJ in intrinsic UPJO compared to normal subjects and chronic UPJO.
Lee et al., Korea (37)	8: 8	37 to 54 years age range	Two groups of specimens were studied: proximal group ≤ 5 cm from the UPJ, distal group ≥5 cm from UPJ. IHC was performed on the obtained tissues and observed under 400X magnification. Contractibility was assessed based on the dose dependent response of acetylcholine and norepinephrine.	Between inner longitudinal muscles and interface between inner longitudinal and outer circular muscle layers	CLCs were found abundantly in the proximal group. There were spontaneous contractions (3 to 4 contractions within 5 min) in the proximal group. Distal sections did not show any spontaneous contractions.	No CLCs were found in the distal group.	Spontaneous contractions in human ureter could be generated by CLCs in the proximal region. This action might be regulated by cholinergic and/or adrenergic systems.
Mehrazma et al., Iran (38)	25: 19	1.7 (0.1 to 8) years	CLC density at the UPJ in patients with UPJO and controls was compared using IHC (400X magnification)	Between the muscle layers	Mean CLC density per HPF was 14.5 (SD = 5.6)	Mean CLC density per HPF was 32.8 (SD = 11.9)	CLCs density was significantly low in cases compared to controls (*P* < 0.001).
Metzger et al., Germany (39)	56 ureter samples	Cadavers aged 54 (24–82) yearsand patients with renal tumours aged 49 (42–64) years	Samples were obtained from renal pelvis, and proximal, intermediate, and distal ureter. CLC density was assessed following IHC per HPF (200X magnification).	Lamina propria and muscularis propria	CLC density per HPF were: pelvis 13 (range 0.33 to 3.66) proximal ureter 10 (range 0 to 3.00) intermediate ureter 32 (range 0 to 6.66) distal ureter 22 (range 0.33 to 7.66).		CLC density was lower in the proximal ureter compared to the renal pelvis. The CLC density increased from proximal to intermediate ureter, and then decreased at the distal ureter.
Pande et al., India (40)	30: 7	0.7 (0.2–8) years: 2 (0.7–5) years	CLC density at the UPJ in patients with UPJO and controls was compared using IHC (under 400X magnification). Surgical outcome was assessed using ultrasonographs at 6-month post-operatively.	Not available	CLC density in cases per HPF was 4.86 (SD = 0.76)	CLC density in controls per HPF was 11.74 (SD = 0.86)	CLC density was significantly low in cases compared to controls (*p* = 0.04).
Prisca et al., Romania (41)	13	0.6 to 83 years	Samples were obtained from multiple areas of the urinary tract from the deceased with no evidence of UPJO. The obtained samples were categorized into following levels: upper urinary tract: 1st level- Kidney, 2nd level- Calyces, 3rd level- Pyelon, 4th level- UPJ, 5th level- Proximal ureter, 6th level- Middle ureter, 7th level- Distal ureter. IHC was performed and observed under HPF (400X magnification)	Between muscle layers	Median CLC density per HPF at levels; 2nd level- 6 (4 to 9) 3rd level- 5 (2 to 8) 4th level- 4 (2 to 7) 5th level- 3 (1 to 6) 6th level- 2 (1 to 5) 7th level- 2 (0 to 5)		In normal individuals, CLC density was high in the calyces and pyelon, while CLCs were scanty in the mid and distal ureter.
Senol et al., Turkey (42)	19: 12	116 ± 116 months: 279 ± 312 months	CLC density at the UPJ in patients with UPJO and controls was compared using IHC. CLC density per HPF (400X magnification) was graded as: very few (0 to 3), few (4 to 6) and many (>7) cells.	Closer to the inner longitudinal layer	CLC density in cases per HPF is 2.37 (SD = 2.19) Many – 1 (5.3%) Few – 5 (26.3%) Very few – 13 (68.4%)	CLC density in controls per HPF is 24.5 (SD = 9.73)All individuals had >7 CLCs per HPF.	Compared to controls cases had either no or few CLCs (*p* < 0.0001).
Solaris et al., Ireland (43)	19: 7	2.3 (0.2–12) years: 4.5 (0.9–9) years	CLC density at the UPJ in patients with UPJO and controls was compared using IHC. CLC density per HPF (400X magnification) was graded as: sparse (0 to 1), few (2 to 3), moderate (4 to 8), and many (>8).	Inner border of circular muscle layer	CLCs were sparse or absent (<2 per HPF).	CLC density was >8 per HPF (Grading: “many”).	Patients with UPJO have a lower density of CLCs in the UPJ and renal pelvis compared to controls (*p* < 0.05).
Ven der Aa et al., Belgium (14)	44 (65 tissue samples)	39.7 (1–78) years in males, 16 (1–50) years in females	Tissue samples were obtained from renal pelvis, upper, middle, and lower ureter, vesicoureteral junction, bladder dome, bladder neck and urethra. IHC was performed and observed under HPF (400X and 200X magnifications).	Beneath urothelium and between muscle layers	Values not available	Values not available	CLC density was greater in pyelon compared to ureter. No significant difference in the CLC density was observed between upper, mid, and lower thirds of the ureter or between the longitudinal and circular muscle layers of the ureter.
Wishahi et al., Egypt (44)	7: 5	28 ± 10 years :52 ± 7 years	CLC density at the UPJ, PU and RP in patients with UPJO and controls were compared using IHC and transmission electron microscopy.	Between Muscular layers	CLC density was high in PU, moderate in RP, scanty or absent in UPJ.	CLC density was high in the PU, excess in RP, and moderate in the UPJ.	Patients with UPJO have a lower density of CLCs in the UPJ and renal pelvis compared to controls (*p* < 0.05).
Yang et al., China (45)	24: 21	0.25 to 12 years	CLC density at the UPJ in patients with UPJO and controls was compared using IHC (400X magnification)	Between muscle layers	Density of CLCs per HPF in cases was 0.207 (SD = 0.020).	Density of CLCs per HPF in controls was 0.262 (SD = 0.026).	CLC density at the UPJ was significantly lower in the cases compared to the controls (*p* < 0.05).

### Distribution of Cajal-Like Cells

Majority (11/20, 55%) of the studies found CLCs between the inner longitudinal and outer circular muscle layers or in close proximity to the muscle layers (7/20, 35%), while others found these cells to be present both in the lamina propria and serosal layers (1/20, 5%) in addition to the muscle layers (Supplementary Table 4).

The reported distribution of the CLCs in different parts of the upper urinary system were controversial. Wishahi et al. (44) reported that the CLC density gradually increased from renal pelvis to proximal ureter in healthy subjects, while two studies found a decrease in CLC density from UPJ to distal ureter (37, 41). Conversely, Metzger et al. (39) reported that the CLC density gradually increased from the pelvis to the intermediate ureter, and then reduced at the distal ureter, while Ven Der Aa et al. (14) could not find a statistically significant difference in CLC density between upper, mid and distal thirds of the ureter.

Most studies (31–33, 42, 45) reported a lower density of CLCs in the UPJ of the patients with UPJO compared to the controls (Table 2). On the contrary, Koleda et al. (23), and Kuvel et al. (22) found a comparatively higher density of CLCs at the UPJ in patients. How et al. (34) found no statistically significant difference between the CLC density in the UPJ between the cases and controls. Apoznanski et al. (1) explored the density of CLCs in affected patients with UPJO by quantifying the density of CLCs in adjacent high-power fields of the UPJ and calculated the gradient of CLCs. They found no significant differences of the CLC gradient between cases and controls (1).

### Age Related Changes in Cajal-Like Cells

**Figure 2:**
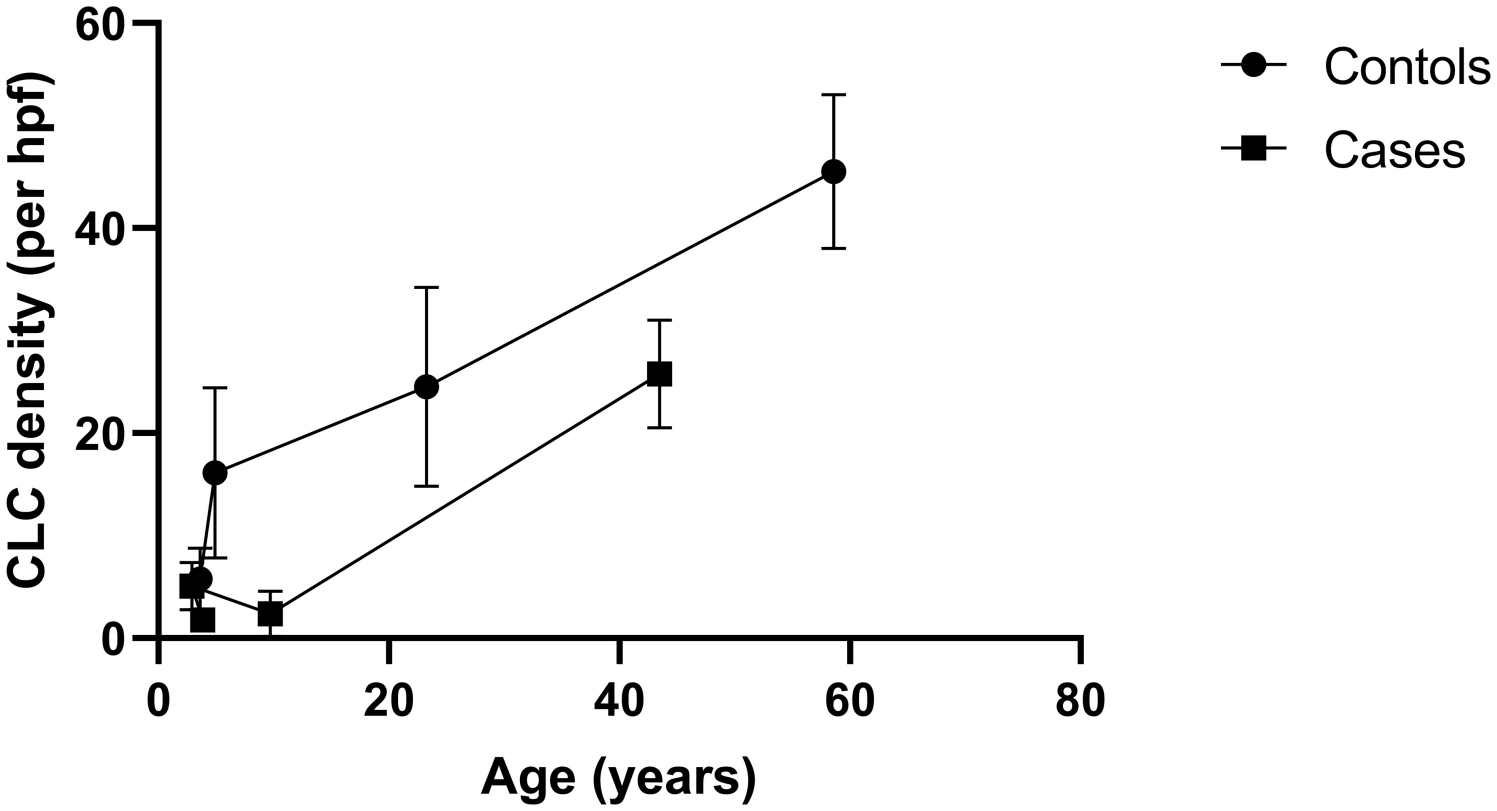
A line diagram demonstrating the associations of density of Cajal like cells (CLCs) at the ureteropelvic junction and age. The diagram illustrates that the CLC density increases with age in both healthy and those affected with ureteropelvic junction obstruction. Data from four studies were used to create the chart (31, 32, 36, 42).

The CLC density increases in the UPJ with the advancement of the gestational age of the fetal ureter (31). Nevertheless according to Koleda et al. (23) the density gradually decrease as the age advanced into childhood. Based on the studies included in the quantitative synthesis, a line diagram was drawn to illustrate the CLC density at the UPJ and we found an increase in CLC density with age in both healthy and those affected with UPJO (31, 32, 36, 42) (Figure 2). Further, the affected subjects consistently had a low CLC density compared to the healthy controls.

### Cajal-Like Cell Contribution to Post-operative Outcome

Two studies exploring the association between the post-operative outcome of Anderson-Hynes pyeloplasty and the CLC density at the resected margin of ureter, showed that patients with a higher density of CLCs had a better surgical functional outcome (15, 35). Nevertheless, Pande et al. (40) found no correlation between the CLC density and the post-operative functional outcome.

### Meta-Analysis

**Figure 3:**
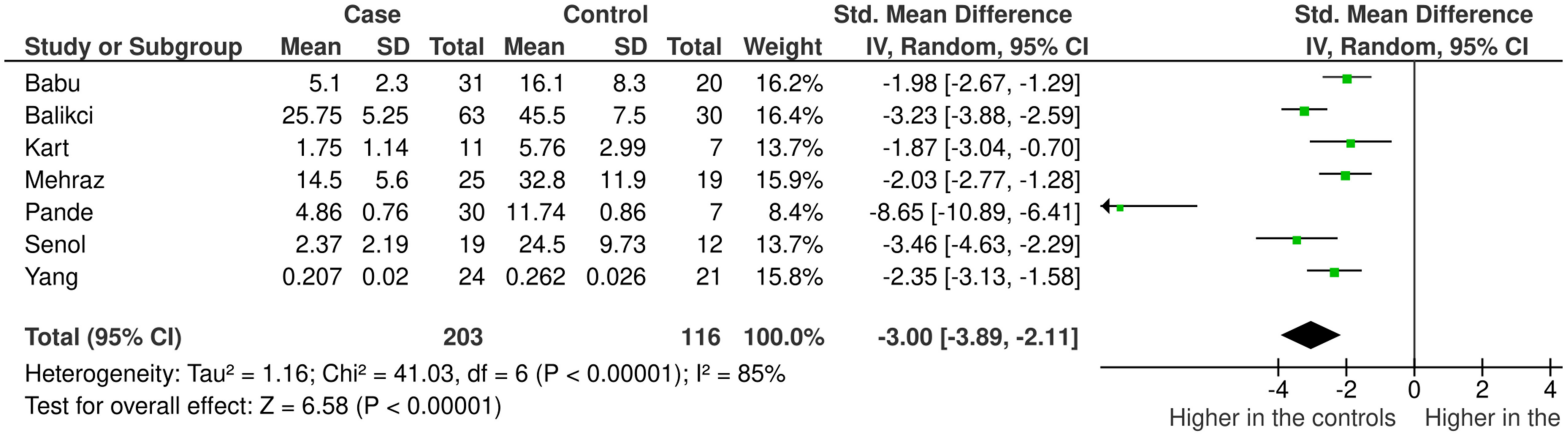
The study characteristics and standardized mean differences of the density of the interstitial cells of Cajal like cells at the ureteropelvic junction in patients with ureteropelvic junction obstruction and matched controls.

Seven studies reporting the mean difference of the density of CLC in the UPJ per high power field in patient with UPJO and controls were included in the meta-analysis. In the pooled analysis, the density of CLCs was significantly low in patients with UPJO (standardized mean difference = −3.00, 95% confidence interval = −3.89 to −2.11, *p* < 0.01) (Figure 3). The funnel plot of the selected studies is provided in the Supplementary Figure 1. We detected a considerable heterogeneity in this comparison (χ^2^ = 41.03, *I*^2^ = 85%, *df* = 6, *p* < 0.01). We performed a sensitivity analysis by including studies conducted on children only (aged <14 years) (*n* = 5) (31, 36, 38, 40, 45). The studies including both children (<2 years) and elders (>70 years) were excluded (32, 42). Nonetheless, the sensitivity analysis found a standardized mean difference of −2.93 (95% CI = −4.14 to −1.73) with a considerable heterogeneity (χ^2^ = 32.71, *I*^2^ = 88%, df = 4, *p* < 0.01) (Supplementary Figure 2). We were unable to perform a subgroup analysis comparing pediatric and adult populations since none of the included studies had a homogeneous adult population. To explore the effect of sample sizes, we performed another sensitivity analysis after including studies with at least 10 samples per group (*n* = 5) (31, 32, 38, 42, 45). The results showed a standardized mean difference of −2.56 (95% CI = −3.14 to −1.97) with a substantial heterogeneity (χ^2^ = 11.40, *I*^2^ = 65%, df = 4, *p* < 0.01) (Supplementary Figure 3). Subsequently, we combined the two sensitivity analyses by including studies of children with a large sample size (as defined above) (*n* = 3) (31, 38). In this analysis we found a standardized mean difference of −2.11 (95% CI= −2.53 to −1.68) with no heterogeneity (χ^2^ = 0.56, *I*^2^ = 0%, *df* = 2, *p* < 0.01) (Supplementary Figure 4).

## Discussion

UPJO is the partial or intermittent blockage of urinary flow from the renal pelvis into the ureter, governed by either an anatomical derangement or in most instances a functional disturbance (5, 26, 46–48). About a decade ago, dilemma on the pathophysiology of UPJO brought myogenic theory to light, which suggests that uncoordinated muscular contractions at the UPJ leads to a functional obstruction of antegrade urine flow (49). The discovery of CLCs in the upper urinary tract which could propagate action potentials in the UPJ, intrigued researchers to investigate into their role in UPJO (49).

### Cajal Like Cells in the Upper Urinary Tract

Ureteric wall consists of a transitional epithelium, lamina propria, inner longitudinal, and outer circular muscle layers and a serosa. In most studies, CLCs were located between the inner longitudinal and outer circular muscle layers (Supplementary Table 4). Few studies found CLCs in the lamina propria (14, 22, 39), while a single study detected CLCs in the serosa (22). Cajal cells in the intestines, are located near the myenteric plexus and submucosal plexus, between longitudinal and circular muscle layers, between inner and outer circular muscle layers and within interlamellar connective tissues of circular muscles (50). They are, however not often observed in serosa. Similarly, in ureter, CLCs are not readily located in the serosa in most instances but are present considerably more in the lamina propria. These cells are believed to play a coordinator role of impulse transmission between the sensory nerve endings and smooth muscle cells (18, 19), hence are located in areas richly innervated by sensory nerves. Ureteric innervation is to the muscular and subepithelial layers (51) where the nerve endings reside, therefore the deficiency in CLCs in serosal layer could be due to the lack of sensory nerve endings in the serosa.

Majority of the studies suggest that the overall CLC density at UPJ is reduced in individuals with UPJO compared to controls (33, 38, 40, 42, 45), which is consistent with the results of our quantitative synthesis. A constellation of gastrointestinal motility diseases including achalasia cardia (52) and Hirschsprung's disease (53) are associated with depletion of Cajal cells, while reduction of Cajal cell density in small intestinal segments of inflammation or obstruction significantly improves when treating the pathology causing inflammation or removal of obstruction (54, 55). Abstracting from this knowledge, a theory was postulated on the lack of CLCs in the UPJ as a contributor of failed peristaltic wave propagation across the UPJ in UPJO. However, the observational nature of these studies lacked the ability to derive a direct causation, but only an association. This putative role of CLCs was projected to doubt by Koleda et al. (23) and Kuvel et al. (22) with their description of an increase in the CLC density in the UPJ in affected individuals. Interestingly, in Koleda et al. (23) study, the age of the cases was markedly higher compared to the controls which could have contributed to the rising CLCs in cases, since there is a gradual increase of the CLC density with age in normal individuals as well as in patients with UPJO (Figure 2). Similarly, data was lacking on the age of the subjects in Kuvel et al. (22) study. Due to this reason, it may not be prudent to derive meaningful comparisons of the CLC density in cases and controls from the latter two studies. Although CLC density increases in the urinary tract with aging (Figure 2), the Cajal cell number and volume reduces steadily in colon and stomach (56). Furthermore, Cajal cell loss and aging increases slow waves conduction velocity in the stomach (57) resulting in delayed gastric emptying. Nevertheless it is possible that other syncytial factors have an interdependent role with Cajal cells giving rise to slow wave velocity changes (57).

Though immunohistochemical studies have failed to establish differences of the expression levels of neuronal markers in UPJO (34), it is suggested that a defective innervation at the UPJ in intrinsic obstruction could contribute to increase in CLC density causing increased peristaltic activity as a result of an attempt to overcome peristaltic failure (22). In chronic UPJO from tumors or ureteric stones up-regulation of c-kit expression is not observed to overcome the obstruction (22). The excitatory impulses are generated from a single site of origin propelling urine into the ureter (58). However, when more than one impulse generator sites are present, they block the conduction of waves of excitation (58). This suggests that if there is a change in distribution of impulse generating CLCs in UPJ, it may contribute to alteration of impulse generation leading to intrinsic UPJO. This hypothesis is supported by a study conducted on rabbits where increased frequency of spontaneous mechanical activity of the UPJ was observed during obstruction (59). Researchers pondered on the distributional changes in CLCs in the pathogenesis of UPJO, to which Apoznanski et al. (1) answered by demonstrating no distributional gradient changes in the CLCs in UPJO compared to the age of the affected. However, this study included only seven cases. In addition, we noted that there is a marked deficiency in studies that embark on the distributive changes in the CLCs in affected and healthy UPJ.

CLCs do not possess a primary action potential generation ability in animals, but form a conduit for transmission of signals (60) (Animal study findings related to CLCs are summarized in Supplementary Table 5). Guinea pig renal system, which resembles similar anatomy to humans, shows pacemaker oscillations at the pelvicalyceal junction and UPJ, while oscillations are absent in the ureter (19). When the proximal pacemaker drive is blocked either by pharmacological means or by transection, the distal regions take over the waves of excitation (61), suggesting the presence of pacemaker potential generation mechanism in the mid and distal ureter. These findings corroborate the results of the human studies where CLCs, the potential pacemakers of the renal tract, are not restricted to the renal pelvis and UPJ, but also found in the mid and distal ureter to coordinate unidirectional peristaltic activity.

## Limitations

Few studies (23, 33, 34, 43) could not be incorporated in the quantitative synthesis when the CLC density was not presented as a continuous variable with means and standard deviations as summary statistics. The marked variability of the study designs, especially the wide range of age and limited sample sizes contributed to the high heterogeneity of the quantitative synthesis.

## Quality of Evidence

This systematic review followed the standard recommended methodology set out by PRISMA guidelines. Two reviewers independently assessed the studies for potential sources of bias and a standard approach of data extraction was employed, thus reducing the risk of performance bias in the review and data extraction errors. PRISMA checklist of the review is presented in Supplementary Table 6.

## Conclusions

Cajal like cells are predominantly distributed between the muscle layers of the upper urinary tract. However, the distribution of CLCs along the urinary tract from the renal pelvis toward the lower ureter is subjected to controversy. The CLC density at the UPJ is significantly low in patients with UPJO compared to the controls, suggesting a pivotal contribution by CLCs in the pathogenesis of UPJO. The CLC density gradually increases with aging in both healthy subjects and patients with UPJO, which could potentially bias the results of the anatomical studies when age is not strictly matched in cases and controls. Careful matching of age in cases and controls, avoiding large age ranges and using an adequate sample size are necessary when performing future studies.

## Data Availability Statement

The original contributions presented in the study are included in the article/Supplementary Material, further inquiries can be directed to the corresponding authors.

## Author Contributions

US conceptualized the study. YM developed the search strategy. US, YM, and AM extracted data. YM conducted the meta-analysis. US, YM, UL, and AM wrote the first draft of the manuscript. All authors were involved in drafting and commenting on the paper and have approved the final version.

## Conflict of Interest

The authors declare that the research was conducted in the absence of any commercial or financial relationships that could be construed as a potential conflict of interest.

## Publisher's Note

All claims expressed in this article are solely those of the authors and do not necessarily represent those of their affiliated organizations, or those of the publisher, the editors and the reviewers. Any product that may be evaluated in this article, or claim that may be made by its manufacturer, is not guaranteed or endorsed by the publisher.
