# Supplementary material for: Variations in the Density and Distribution of Cajal Like Cells Associated With the Pathogenesis of Ureteropelvic Junction Obstruction: A Systematic Review and Meta-Analysis
Source: Front Surg. 2021 Jul 28;8:721143. doi: 10.3389/fsurg.2021.721143 (PMC8355516; doi:10.3389/fsurg.2021.721143)
Supplement: Supplementary file 1 [file Data_Sheet_1.pdf]

Supplementary Material

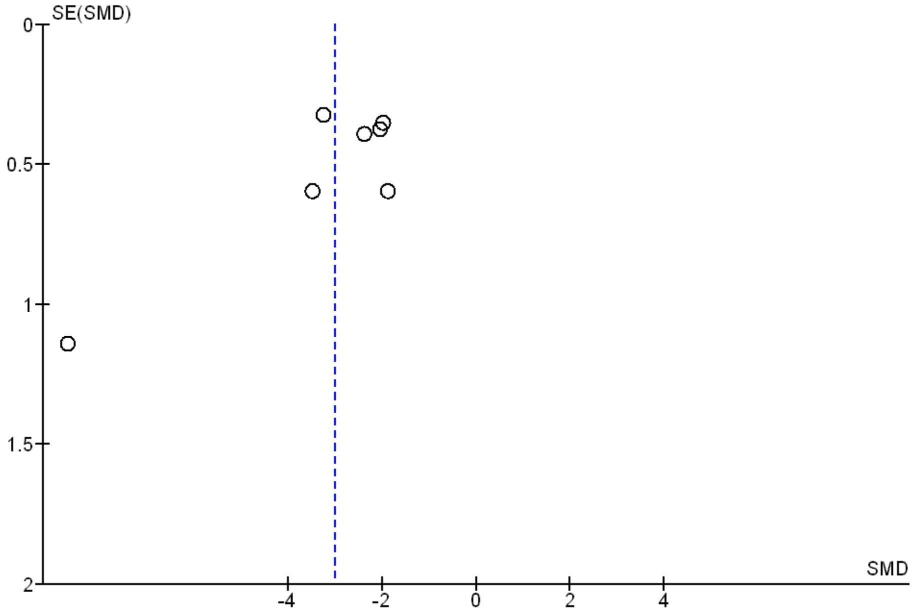

Supplementary Figure 1: The funnel plot of the studies selected for the quantitative synthesis.

	Case			Control				Std. Mean Difference
**Study or Subgroup**	**Mean**	**SD**	**Total**	**Mean**	**SD**	**Total**	**Weight**	**IV, Random, 95% CI**
Babu	5.1	2.3	31	16.1	8.3	20	22.6%	-1.98 [-2.67, -1.29]
Balikci	25.75	5.25	63	45.5	7.5	30	0.0%	-3.23 [-3.88, -2.59]
Kart	1.75	1.14	11	5.76	2.99	7	19.8%	-1.87 [-3.04, -0.70]
Mehraz	14.5	5.6	25	32.8	11.9	19	22.3%	-2.03 [-2.77, -1.28]
Pande	4.86	0.76	30	11.74	0.86	7	13.2%	-8.65 [-10.89, -6.41]
Senol	2.37	2.19	19	24.5	9.73	12	0.0%	-3.46 [-4.63, -2.29]
Yang	0.207	0.02	24	0.262	0.026	21	22.1%	-2.35 [-3.13, -1.58]
**Total (95% CI)**			**121**			**74**	**100.0%**	**-2.93 [-4.14, -1.73]**

Heterogeneity: $Tau^2 = 1.56$; $Chi^2 = 32.71$, df = 4 ($P < 0.00001$); $I^2 = 88\%$

Test for overall effect: Z = 4.77 ($P < 0.00001$)

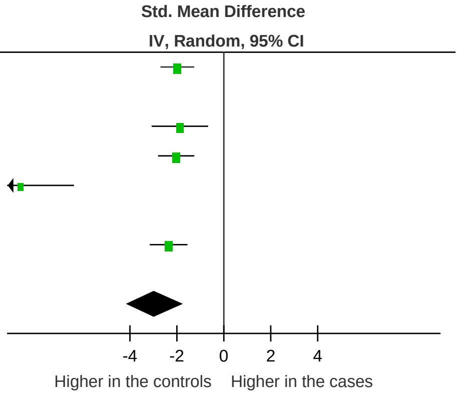

Supplementary Figure 2: Forrest plot of the density of Cajal-like cells at the ureteropelvic junction in studies conducted on children only

	Case			Control				Std. Mean Difference
**Study or Subgroup**	**Mean**	**SD**	**Total**	**Mean**	**SD**	**Total**	**Weight**	**IV, Random, 95% CI**
Babu	5.1	2.3	31	16.1	8.3	20	21.9%	-1.98 [-2.67, -1.29]
Balikci	25.75	5.25	63	45.5	7.5	30	22.8%	-3.23 [-3.88, -2.59]
Kart	1.75	1.14	11	5.76	2.99	7	0.0%	-1.87 [-3.04, -0.70]
Mehraz	14.5	5.6	25	32.8	11.9	19	20.9%	-2.03 [-2.77, -1.28]
Pande	4.86	0.76	30	11.74	0.86	7	0.0%	-8.65 [-10.89, -6.41]
Senol	2.37	2.19	19	24.5	9.73	12	14.0%	-3.46 [-4.63, -2.29]
Yang	0.207	0.02	24	0.262	0.026	21	20.3%	-2.35 [-3.13, -1.58]
**Total (95% CI)**			**162**			**102**	**100.0%**	**-2.56 [-3.14, -1.97]**

Heterogeneity: Tau² = 0.28; Chi² = 11.40, df = 4 (P = 0.02); I² = 65%
Test for overall effect: Z = 8.55 (P < 0.00001)

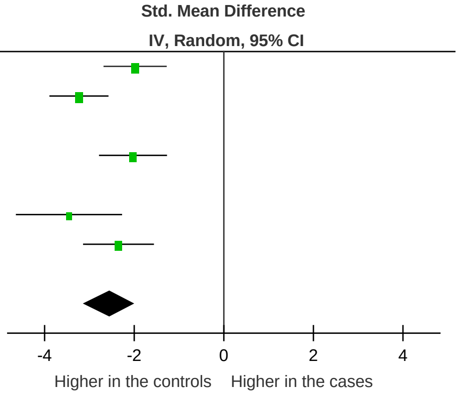

Supplementary Figure 3: Forrest plot of the density of Cajal-like cells at the ureteropelvic junction in studies with a sample size of more than 10 in each group.

	Case			Control				Std. Mean Difference
**Study or Subgroup**	**Mean**	**SD**	**Total**	**Mean**	**SD**	**Total**	**Weight**	**IV, Random, 95% CI**
Babu	5.1	2.3	31	16.1	8.3	20	37.7%	-1.98 [-2.67, -1.29]
Balikci	25.75	5.25	63	45.5	7.5	30	0.0%	-3.23 [-3.88, -2.59]
Kart	1.75	1.14	11	5.76	2.99	7	0.0%	-1.87 [-3.04, -0.70]
Mehraz	14.5	5.6	25	32.8	11.9	19	32.5%	-2.03 [-2.77, -1.28]
Pande	4.86	0.76	30	11.74	0.86	7	0.0%	-8.65 [-10.89, -6.41]
Senol	2.37	2.19	19	24.5	9.73	12	0.0%	-3.46 [-4.63, -2.29]
Yang	0.207	0.02	24	0.262	0.026	21	29.8%	-2.35 [-3.13, -1.58]
**Total (95% CI)**			**80**			**60**	**100.0%**	**-2.11 [-2.53, -1.68]**

Heterogeneity: Tau² = 0.00; Chi² = 0.56, df = 2 (P = 0.76); I² = 0%
Test for overall effect: Z = 9.74 (P < 0.00001)

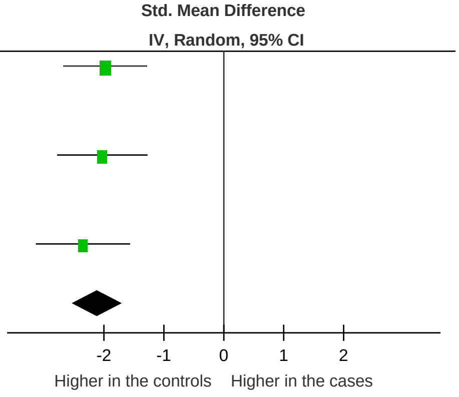

Supplementary Figure 4: Forrest plot of the density of Cajal-like cells at the ureteropelvic junction in studies conducted on children only with a sample size of more than 10 in each group.

Supplementary Table 1: The reasons for excluding articles from the quantitative synthesis

**Study**	**Reason for exclusion from quantitative synthesis**
Apoznanski et al., (2013)	CLC gradient was analyzed instead of the density of the CLCs.
Babu et al., (2020)	No control group for CLC density comparison
Eken et al., (2013)	The density of CLCs was given as an ordinal variable
How et al., (2018)	The density of CLCs was given as an ordinal variable
Inugala et al., (2017)	No control group for CLC density comparison
Koleda et al., (2012)	The density of CLCs was given as an ordinal variable
Kuvel et al., (2011)	No control group for CLC density comparison
Lee et al., (2011)	Data were not available for quantitative analysis
Metzger et al., (2004)	No control group for CLC density comparison
Prisca et al., (2014)	No control group for CLC density comparison
Solaris et al., (2003)	The density of CLCs was given as an ordinal variable
Ven der Aa et al., (2004)	Data not available for quantitative analysis.
Wishahi et al., (2020)	The density of CLCs was given as an ordinal variable

Abbreviations: CLC – Cajal like cell

Supplementary Table 2: Quality assessment tool for case-control studies

**Joanna Briggs Institute (JBI) critical appraisal checklist for case-control studies**													
**Study**	**1**	**2**	**3**	**4**	**5**	**6**	**7**	**8**	**9**	**10**	**Total score**	**Quality rating**	**Include/ Exclude**
Apoznanski et al., (2013)	Y	Y	Y	Y	Y	Y	U	Y	Y	Y	9/10 (90%)	Good	I
Babu et al., (2019)	Y	Y	Y	Y	Y	Y	Y	Y	Y	Y	10/10 (100%)	Good	I
Balikci et al., (2015)	Y	U	Y	Y	U	Y	Y	Y	Y	Y	8/10 (80%)	Good	I
Eken et al., (2013)	Y	U	Y	Y	Y	Y	U	Y	Y	Y	8/10 (80%)	Good	I
How et al., (2018)	Y	Y	Y	U	U	Y	Y	Y	Y	Y	8/10 (80%)	Good	I
Inugala et al., (2017)	Y	N	Y	Y	Y	Y	Y	Y	Y	Y	9/10 (90%)	Good	I
Kart et al., (2013)	Y	Y	Y	Y	Y	Y	Y	Y	Y	Y	10/10 (100%)	Good	I
Koleda et al., (2012)	Y	N	Y	Y	Y	Y	Y	Y	Y	Y	9/10 (90%)	Good	I
Lee et al., (2011)	Y	N	Y	Y	Y	U	U	Y	Y	Y	7/10 (70%)	Good	I
Mehrazma et al., (2014)	Y	Y	Y	Y	Y	Y	Y	Y	Y	Y	10/10 (100%)	Good	I
Pande et at., (2020)	Y	Y	Y	Y	Y	Y	Y	Y	Y	Y	10/10 (100%)	Good	I

Senol et al., (2016)	Y	Y	Y	Y	Y	Y	Y	Y	Y	Y	10/10 (100%)	Good	I
Solaris et al., (2003)	Y	Y	Y	U	Y	Y	Y	Y	Y	Y	9/10 (90%)	Good	I
Wishahi et al., (2020)	Y	Y	Y	Y	Y	Y	Y	Y	Y	U	9/10 (90%)	Good	I
Yang et al., (2009)	Y	Y	Y	Y	Y	Y	Y	Y	Y	Y	10/10 (100%)	Good	I

Quality of the included case-control studies were assessed using Joanna Briggs Institute (JBI) critical appraisal checklist for case-control studies (Moola et al., 2017)**;**

1.Were the groups comparable other than the presence of disease in cases or the absence of disease in controls?

2. Were cases and controls matched appropriately?

3. Were the same criteria used for identification of cases and controls?

4. Was exposure measured in a standard, valid and reliable way?

5. Was exposure measured in the same way for cases and controls?

6. Were confounding factors identified?

7. Were strategies to deal with confounding factors stated?

8. Were outcomes assessed in a standard, valid and reliable way for cases and controls?

9. Was the exposure period of interest long enough to be meaningful?

10. Was appropriate statistical analysis used?

Answers: Yes, No, Unclear, Not applicable: Y, N, U, NA

Total score: number of Yes;

The quality rating: 67-100 (good), 34-66 (fair), and 0-33 (poor).

Include/Exclude: I/ Ex.

Supplementary Table 3: Quality assessment tool for cross-sectional studies

**Briggs Institute (JBI) critical appraisal checklist for analytical cross-sectional studies**											
**Study**	**1**	**2**	**3**	**4**	**5**	**6**	**7**	**8**	**Total score**	**Quality rating**	**Include/ Exclude**
Babu et al., (2020)	Y	Y	Y	Y	Y	U	Y	Y	7/8 (88%)	Good	I
Kuvel et al., (2011)	Y	Y	Y	Y	U	U	Y	Y	6/8 (75%)	Good	I
Metzger et al., (2004)	Y	Y	Y	Y	Y	Y	Y	Y	8/8 (100%)	Good	I
Prisca et al., (2014)	Y	Y	U	Y	Y	Y	Y	Y	7/8 (88%)	Good	I
Ven der Aa et al., (2004)	Y	N	Y	Y	Y	Y	Y	U	6/8 (75%)	Good	I

Quality of the included cross-sectional studies were assessed using Joanna Briggs Institute (JBI) critical appraisal checklist for analytical cross-sectional studies (Moola et al., 2017)**:**

1. Were the criteria for inclusion in the sample clearly defined?

2. Were the study subjects and the setting described in detail?

3. Was the exposure measured in a valid and reliable way?

4. Were objective, standard criteria used for measurement of the condition?

5. Were confounding factors identified?

6. Were strategies to deal with confounding factors stated?

7. Were the outcomes measured in a valid and reliable way?

8. Was appropriate statistical analysis used?

Total Score: Number of yes; CD, cannot be determined; NA, not applicable; NR, not reported; N, no; Y, yes.

Quality Rating: Poor <50%, Fair 50-75%, Good >75%

Supplementary Table 4: The distribution of Cajal-like cells in each layer of the urinary tract

	Between muscle layers	Lamina propria and muscle layers	Serosa	Not given
Apoznanski et al., (2013)		X		
Babu et al., (2020)				X
Babu et al., (2019)	X			
Balikci et al., (2015)		X		
Eken et al., (2013)		X		
How et al., (2018)				X
Inugala et al., (2017)				X
Kart et al., (2013)	X			
Koleda et al., (2012)				X
Kuvel et al., (2011)	X	X	X	
Lee et al., (2011)	X			
Mehrazma et al., (2014)	X			
Metzger et al., (2004)	X	X		
Pande et at., (2020)				X
Prisca et al., (2014)	X			
Senol et al., (2016)		X		

Solaris et al., (2003)	X			
Ven der Aa et al., (2004)	X	X		
Wishahi et al., (2020)	X			
Yang et al., (2009)	X			
**TOTAL**	11	7	1	5

Supplementary Table 5: A summary of animal studies assessing the role of Cajal-like cells in ureteropelvic junction obstruction.

Author (year)	Type of animal	Method of collection and identification of CLCs	Research findings	Conclusions
Klemm et al., (1999)	Guinea-pig	upper urinary tract was examined electrophysiologically using intracellular microelectrodes, morphologically using electron and confocal microscopy	Pacemaker oscillations were recorded at pelvicalyceal junction (83% cells) and proximal renal pelvis (15% cells), but not in distal renal pelvis and ureter.<br><br>Spontaneous action potentials were generated at proximal renal pelvis (75%), distal renal pelvis (89%) and ureter (100%).<br><br>Spontaneously discharging CLCs were seen in lamina propria of renal pelvis and pelvicalyceal junction.	Atypical smooth muscles cells generate pacemaker potentials, while CLCs amplify pacemaker signals to initiate action potentials in the upper urinary tract.
Hashitani et al., (2017)	Mouse	CLCs at the renal pelvis were examined with Focused Ion Beam milling combined with Scanning Electron Microscopy (FIB-SEM), IHC and Ca^{2+} imaging.	CLCs were present in the adventitia and the suburothelial space adjacent to typical and atypical smooth muscle cells.<br><br>CLCs had spontaneous low-frequency, asynchronous Ca2+ transients that synchronized<br><br>into a burst every 3–5 min. Atypical smooth muscle cells showed higher-frequency spontaneous Ca2+ transients and accelerated behavior in synchrony with the bursts of CLCs.	CLCs generate a slow voltage-dependent pyelo-ureteric excitability. They do not represent the primary pacemaker, rather act as an accelerator of the atypical smooth muscle derived<br><br>pacemaker drive.
Kuzgunbay et al., (2009)	Rat	Controls (20), sham operation (20), study group (69).	Mean density of CLCs in controls and cases were 4.55 (SD=2.21) and 5.15 (SD=3.51), respectively. There was no significant change of the	CLC density increases following

		Study group underwent distal ureteric ligature close to the vesicoureteric junction. All cases underwent nephrectomy at 7, 14, 30, 60 and 90 days following ligature. The UPJs were studied using IHC.	CLC density at the UPJ over time in rats undergoing sham surgery and controls. Following ligature at the vesico-ureteric junction, the study group had an increased CLC density at the UPJ with time. Maximum increase in CLC density was observed 14 days following placing the ligature.	distal ureteric obstruction.
Metzger et al., (2005)	Rodents, porcine, carnivores, cow and humans	CLC density at renal pelvis and ureteral specimens obtained from humans, rodents, porcine, carnivores and cow were compared using IHC.	CLC density was highest in the pyelon in both humans and pigs. The other animals had variable distribution of CLCs in their urinary tract.	Pigs have similar CLC distribution in the ureter compared to humans.
Metzger et al., (2008)	Pig	CLC density at renal calices, renal pelvis, UPJ, proximal, middle and distal ureter, ureteral orifice, bladder and urethra was assessed using IHC.	The highest density of CLCs was observed at UPJ. Nevertheless, the differences of CLC density between the segments were minimal. CLCs were arranged parallelly to the smooth muscle cell layers.	The close relationship of CLCs with smooth muscle cells suggest a contribution to the intrinsic pacemaker activity.

Abbreviations: CLCs - Cajal like cells, FIB SEM - focused ion beam scanning electron microscopy, IHC - Immunohistochemistry, UPJ - ureteropelvic junction, UPJO - ureteropelvic junction obstruction,

Supplementary Table 6: PRISMA checklist

		Checklist item	Page #
**TITLE**			
Title	1	Identify the report as a systematic review, meta-analysis, or both.	1
**ABSTRACT**			
Structured summary	2	Provide a structured summary including, as applicable: background; objectives; data sources; study eligibility criteria, participants, and interventions; study appraisal and synthesis methods; results; limitations; conclusions and implications of key findings; systematic review registration number.	1
**INTRODUCTION**			
Rationale	3	Describe the rationale for the review in the context of what is already known.	2
Objectives	4	Provide an explicit statement of questions being addressed with reference to participants, interventions, comparisons, outcomes, and study design (PICOS).	2
**METHODS**			
Protocol and registration	5	Indicate if a review protocol exists, if and where it can be accessed (e.g., Web address), and, if available, provide registration information including registration number.	2
Eligibility criteria	6	Specify study characteristics (e.g., PICOS, length of follow-up) and report characteristics (e.g., years considered, language, publication status) used as criteria for eligibility, giving rationale.	2
Information sources	7	Describe all information sources (e.g., databases with dates of coverage, contact with study authors to identify additional studies) in the search and date last searched.	2
Search	8	Present full electronic search strategy for at least one database, including any limits used, such that it could be repeated.	Table 1
Study selection	9	State the process for selecting studies (i.e., screening, eligibility, included in systematic review, and, if applicable, included in the meta-analysis).	2, Figure 1
Data collection process	10	Describe method of data extraction from reports (e.g., piloted forms, independently, in duplicate) and any processes for obtaining and confirming data from investigators.	2
Data items	11	List and define all variables for which data were sought (e.g., PICOS, funding sources) and any assumptions and	2

		simplifications made.	
Risk of bias in individual studies	12	Describe methods used for assessing risk of bias of individual studies (including specification of whether this was done at the study or outcome level), and how this information is to be used in any data synthesis.	3, Supplementary Tables 2 and 3
Summary measures	13	State the principal summary measures (e.g., risk ratio, difference in means).	Table 2
Synthesis of results	14	Describe the methods of handling data and combining results of studies, if done, including measures of consistency (e.g., I^2) for each meta-analysis.	8
Risk of bias across studies	15	Specify any assessment of risk of bias that may affect the cumulative evidence (e.g., publication bias, selective reporting within studies).	N/A
Additional analyses	16	Describe methods of additional analyses (e.g., sensitivity or subgroup analyses, meta-regression), if done, indicating which were pre-specified.	8
**RESULTS**			
Study selection	17	Give numbers of studies screened, assessed for eligibility, and included in the review, with reasons for exclusions at each stage, ideally with a flow diagram.	Figure 1, Supplementary Table 1
Study characteristics	18	For each study, present characteristics for which data were extracted (e.g., study size, PICOS, follow-up period) and provide the citations.	Table 2
Risk of bias within studies	19	Present data on risk of bias of each study and, if available, any outcome level assessment (see item 12).	Supplementary Tables 2, 3
Results of individual studies	20	For all outcomes considered (benefits or harms), present, for each study: (a) simple summary data for each intervention group (b) effect estimates and confidence intervals, ideally with a forest plot.	Figure 3
Synthesis of results	21	Present results of each meta-analysis done, including confidence intervals and measures of consistency.	3, Figure 3, Supplementary Figure 1
Risk of bias across studies	22	Present results of any assessment of risk of bias across studies (see Item 15).	NA
Additional analysis	23	Give results of additional analyses, if done (e.g., sensitivity or subgroup analyses, meta-regression [see Item 16]).	Supplementary Figures 2-4
**DISCUSSION**			
Summary of evidence	24	Summarize the main findings including the strength of evidence for each main outcome; consider their relevance to key groups (e.g., healthcare providers, users, and policy	8-9

		makers).	
Limitations	25	Discuss limitations at study and outcome level (e.g., risk of bias), and at review-level (e.g., incomplete retrieval of identified research, reporting bias).	10
Conclusions	26	Provide a general interpretation of the results in the context of other evidence, and implications for future research.	10
**FUNDING**			
Funding	27	Describe sources of funding for the systematic review and other support (e.g., supply of data); role of funders for the systematic review.	10

From: Moher, D., Liberati, A., Tetzlaff, J., Altman, D. G., and PRISMA Group (2009). Preferred reporting items for systematic reviews and meta-analyses: the PRISMA statement. *PLoS medicine, 6*(7), e1000097.

References

Apoznanski, W., Koleda, P., Wozniak, Z., Rusiecki, L., Szydelko, T., Kalka, D., and Pilecki, W. (2013). The distribution of interstitial cells of Cajal in congenital ureteropelvic junction obstruction. *International urology and nephrology* 45**,** 607-612.

Babu, R., Vittalraj, P., Sundaram, S., Manjusha, M.P., Ramanan, V., and Sai, V. (2020). Comparison of different pathological markers in predicting pyeloplasty outcomes in children. *Journal of pediatric surgery* 55**,** 1616-1620.

Babu, R., Vittalraj, P., Sundaram, S., and Shalini, S. (2019). Pathological changes in ureterovesical and ureteropelvic junction obstruction explained by fetal ureter histology. *Journal of pediatric urology* 15**,** 240. e241-240. e247.

Balikci, Ö., Turunç, T., Bal, N., Çelik, H., and Özkardeş, H. (2015). Comparison of Cajal-like cells in pelvis and proximal ureter of kidney with and without hydronephrosis. *International braz j urol* 41**,** 1178-1184.

Eken, A., Erdogan, S., Kuyucu, Y., Seydaoglu, G., Polat, S., and Satar, N. (2013). Immunohistochemical and electron microscopic examination of Cajal cells in ureteropelvic junction obstruction. *Canadian Urological Association Journal* 7**,** E311.

Hashitani, H., Nguyen, M.J., Noda, H., Mitsui, R., Higashi, R., Ohta, K., Nakamura, K.-I., and Lang, R.J. (2017). Interstitial cell modulation of pyeloureteric peristalsis in the mouse renal pelvis examined using FIBSEM tomography and calcium indicators. *Pflügers Archiv-European Journal of Physiology* 469**,** 797-813.

How, G.Y., Chang, K.T.E., Jacobsen, A.S., Yap, T.-L., Ong, C.C.P., Low, Y., Allen, J.C., Kuick, C.H., Lim, M.Z.L., and Laksmi, N.K. (2018). Neuronal defects an etiological factor in congenital pelviureteric junction obstruction? *Journal of Pediatric Urology* 14**,** 51. e51-51. e57.

Inugala, A., Reddy, R.K., Rao, B.N., Reddy, S.P., Othuluru, R., Kanniyan, L., Kumbha, N., and Srirampur, S. (2017). Immunohistochemistry in ureteropelvic junction obstruction and its correlation to postoperative outcome. *Journal of Indian Association of Pediatric Surgeons* 22**,** 129.

Kart, Y., Karakuş, O.Z., Ateş, O., Hakgüder, G., Olguner, M., and Akgür, F.M. (2013). Altered expression of interstitial cells of Cajal in primary obstructive megaureter. *Journal of pediatric urology* 9**,** 1028-1031.

Klemm, M.F., Exintaris, B., and Lang, R.J. (1999). Identification of the cells underlying pacemaker activity in the guinea-pig upper urinary tract. *The Journal of Physiology* 519**,** 867-884.

Koleda, P., Apoznanski, W., Wozniak, Z., Rusiecki, L., Szydelko, T., Pilecki, W., Polok, M., Kalka, D., and Pupka, A. (2012). Changes in interstitial cell of Cajal-like cells density in congenital ureteropelvic junction obstruction. *International urology and nephrology* 44**,** 7-12.

Kuvel, M., Canguven, O., Murtazaoglu, M., and Albayrak, S. (2011). Distribution of Cajal like cells and innervation in intrinsic ureteropelvic junction obstruction. *Società Italiana***,** 116.

Kuzgunbay, B., Doran, F., Bayazit, Y., Turunc, T., Satar, N., and Kayis, A.A. (2009). The effects of ureteral obstruction on Cajal-like cells in rats. *Journal of pediatric urology* 5**,** 269-273.

Lee, H.W., Baak, C.H., Lee, M.Y., and Kim, Y.C. (2011). Spontaneous contractions augmented by cholinergic and adrenergic systems in the human ureter. *The Korean Journal of Physiology & Pharmacology* 15**,** 37-41.

Mehrazma, M., Tanzifi, P., and Rakhshani, N. (2014). Changes in structure, interstitial Cajal-like cells and apoptosis of smooth muscle cells in congenital ureteropelvic junction obstruction. *Iranian Journal of Pediatrics* 24**,** 105.

Metzger, R., Neugebauer, A., Rolle, U., Böhlig, L., and Till, H. (2008). C-Kit receptor (CD117) in the porcine urinary tract. *Pediatric surgery international* 24**,** 67-76.

Metzger, R., Schuster, T., Till, H., Franke, F.-E., and Dietz, H.-G. (2005). Cajal-like cells in the upper urinary tract: comparative study in various species. *Pediatric surgery international* 21**,** 169-174.

Metzger, R., Schuster, T., Till, H., Stehr, M., Franke, F.-E., and Dietz, H.-G. (2004). Cajal-like cells in the human upper urinary tract. *The Journal of urology* 172**,** 769-772.

Moola, S., Munn, Z., Tufanaru, C., Aromataris, E., Sears, K., Sfetcu, R., Currie, M., Qureshi, R., Mattis, P., and Lisy, K. (2017). Chapter 7: Systematic reviews of etiology and risk. *Joanna Briggs Institute Reviewer's Manual. The Joanna Briggs Institute***,** 2019-2005.

Pande, T., Dey, S.K., Chand, K., and Kinra, P. (2020). Influence of interstitial cells of cajal in congenital ureteropelvic junction obstruction. *Journal of Indian Association of Pediatric Surgeons* 25**,** 231.

Prișcă, R.-A., Loghin, A., Gozar, H.-G., Moldovan, C., Mosó, T., Derzsi, Z., and Borda, A. (2014). Morphological aspects and distribution of interstitial cells of Cajal in the human upper urinary tract. *Turkish Journal of Pathology* 30**,** 100-104.

Senol, C., Onaran, M., Gurocak, S., Gonul, I.I., and Tan, M.O. (2016). Changes in Cajal cell density in ureteropelvic junction obstruction in children. *Journal of Pediatric Urology* 12**,** 89. e81-89. e85.

Solari, V., Piotrowska, A.P., and Puri, P. (2003). Altered expression of interstitial cells of Cajal in congenital ureteropelvic junction obstruction. *The Journal of urology* 170**,** 2420-2422.

Van Der Aa, F., Roskams, T., Blyweert, W., Ost, D., Bogaert, G., and De Ridder, D. (2004). Identification of kit positive cells in the human urinary tract. *The Journal of urology* 171**,** 2492-2496.

Wishahi, M., Mehena, A., Elganzoury, H., Badawy, M., Hafiz, E., and El-Leithy, T. (2020). Telocytes and Cajal cells distribution in renal pelvis, ureteropelvic junction (UPJ), and proximal ureter in normal upper urinary tract and UPJ obstruction: reappraisal of the etiology of UPJ obstruction. *Folia Morphologica*.

Yang, X., Zhang, Y., and Hu, J. (2009). The expression of Cajal cells at the obstruction site of congenital pelviureteric junction obstruction and quantitative image analysis. *Journal of pediatric surgery* 44**,** 2339-2342.
